# NMDA receptor subunit expression and PAR2 receptor activation in colospinal afferent neurons (CANs) during inflammation induced visceral hypersensitivity

**DOI:** 10.1186/1744-8069-5-54

**Published:** 2009-09-22

**Authors:** Shelby K Suckow, Robert M Caudle

**Affiliations:** 1 Department of Neuroscience, University of Florida College of Medicine, Gainesville, FL 32610, USA; 2 Department of Oral and Maxillofacial Surgery and Diagnostic Sciences, University of Florida College of Dentistry, Gainesville, Florida 32610, USA

## Abstract

**Background:**

Visceral hypersensitivity is a clinical observation made when diagnosing patients with functional bowel disorders. The cause of visceral hypersensitivity is unknown but is thought to be attributed to inflammation. Previously we demonstrated that a unique set of enteric neurons, colospinal afferent neurons (CANs), co-localize with the NR1 and NR2D subunits of the NMDA receptor as well as with the PAR2 receptor. The aim of this study was to determine if NMDA and PAR2 receptors expressed on CANs contribute to visceral hypersensitivity following inflammation. Recently, work has suggested that dorsal root ganglion (DRG) neurons expressing the transient receptor potential vanilloid-1 (TRPV1) receptor mediate inflammation induced visceral hypersensitivity. Therefore, in order to study CAN involvement in visceral hypersensitivity, DRG neurons expressing the TRPV1 receptor were lesioned with resiniferatoxin (RTX) prior to inflammation and behavioural testing.

**Results:**

CANs do not express the TRPV1 receptor; therefore, they survive following RTX injection. RTX treatment resulted in a significant decrease in TRPV1 expressing neurons in the colon and immunohistochemical analysis revealed no change in peptide or receptor expression in CANs following RTX lesioning as compared to control data. Behavioral studies determined that both inflamed non-RTX and RTX animals showed a decrease in balloon pressure threshold as compared to controls. Immunohistochemical analysis demonstrated that the NR1 cassettes, N1 and C1, of the NMDA receptor on CANs were up-regulated following inflammation. Furthermore, inflammation resulted in the activation of the PAR2 receptors expressed on CANs.

**Conclusion:**

Our data show that inflammation causes an up-regulation of the NMDA receptor and the activation of the PAR2 receptor expressed on CANs. These changes are associated with a decrease in balloon pressure in response to colorectal distension in non-RTX and RTX lesioned animals. Therefore, these data suggest that CANs contribute to visceral hypersensitivity during inflammation.

## Background

Functional bowel disorders are commonly characterized by altered motility of the gut as well as abdominal pain. Patients with bowel disorders are known to have decreased thresholds to pain for both visceral and somatic stimuli [1]. Ritchie [2] reported that patients with irritable bowel syndrome (IBS) experience colonic pain at lower balloon distension pressures than controls following colorectal distension (CRD). Pain associated with low pressure balloon distension is thought to be indicative of visceral hypersensitivity caused by abnormal motility patterns in patients with bowel disorders. The molecular mechanism that is responsible for visceral hypersensitivity is still currently unknown; however, it is thought to be caused by changes in neuronal excitability of visceral afferents [1].

To date, it is thought that visceral hypersensitivity is mediated by sensory nociceptors of the dorsal root ganglia (DRG) that transmit signals from the gut to the lumbosacral spinal cord. Studies have shown a change in expression of SP and CGRP [3-6] as well as the activation of the NMDA receptor in response to inflammation [7-12] within DRG neurons as well as spinal neurons. However, Zhou et al. [12] reported that the N1 and C1 cassettes of the NMDA receptor NR1 subunit are up-regulated in the colon following 2,4,6-Trinitrobenzenesulfonic acid (TNBS)-induced inflammation. It was hypothesized that this up-regulation is involved in both visceral and somatic hypersensitivity [13,14]. In addition, enteric neurons expressing the proteinase-activated receptor- 2 (PAR2) receptor were found to be involved in visceral hypersensitivity. Coelho et al. [15] demonstrated that by administering the PAR2 specific agonist trypsin hyperalgesia occurred in response to CRD in rats. Furthermore, Cenac et al [16] reported that activation of the PAR2 receptor by proteases generated hypersensitivity symptoms in IBS patients.

Recently we demonstrated that a unique set of enteric neurons, colospinal afferent neurons (CANs), co-localize with several known nociceptive peptides and receptors [17]. The aim of the current study was to determine if CANs contribute to visceral hypersensitivity following TNBS-induced inflammation. To date, studies have shown that the transient receptor potential vanilloid receptor 1 (TRPV1) has a functional role in visceral hypersensitivity [18,19]. In our previous study we demonstrated that the TRPV1 receptor was not expressed in CAN[17]. In the present study we used resiniferatoxin (RTX) to lesion TRPV1 spinal primary afferent innervation to the colon to determine if the lesion could suppress hypersensitivity to CRD.

## Results

### CANs co-localize with neuron specific sodium channel Na_v_1.9

**Figure 1:**
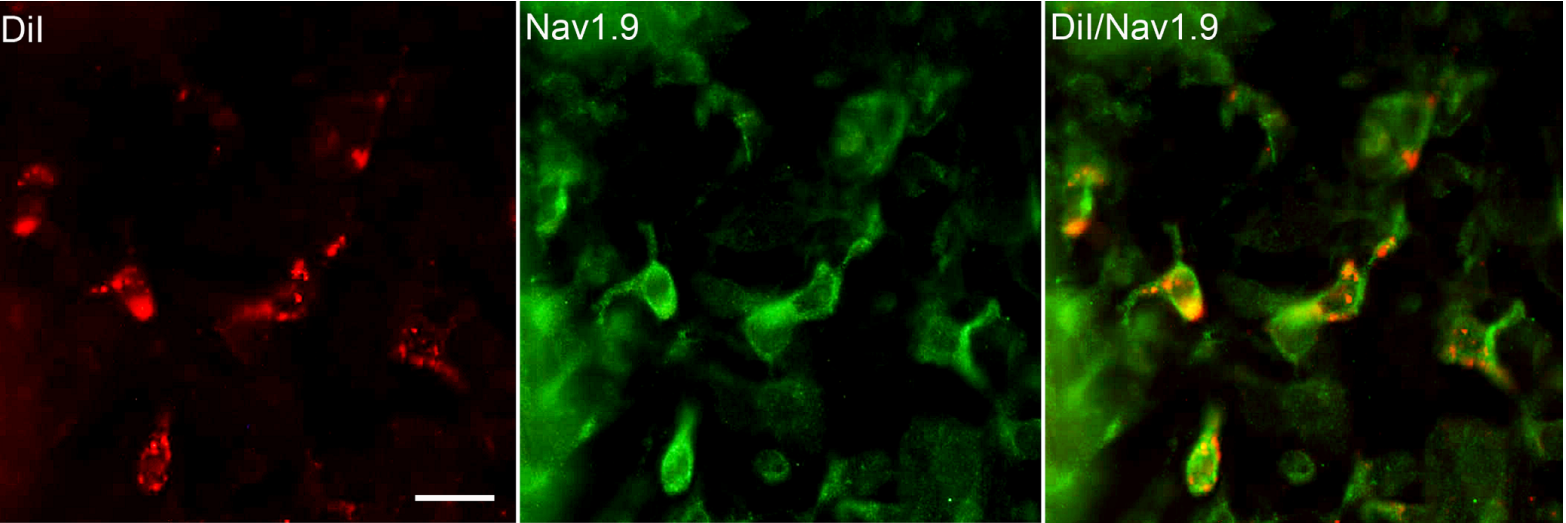
**CANs express TTX-resistant sodium channel Na_v_1.9**. Photomicrographs of DiI labelled neurons in the submucosal plexus of colon showing co-localization with known nociceptive sodium channel Na_v_1.9. Scale bars = 20 μm.

We previously reported that CANs are putative sensory neurons within the enteric nervous system. It was hypothesized that CANs are involved in nociception as demonstrated by immunohistochemical characterization [17]. In order to determine whether CANs are putative nociceptive neurons, we performed immunohistochemistry (n = 5) using an antibody raised against Na_v_1.9. Co-localization demonstrated that 86% of CANs express the sodium channel Na_v_1.9 (Fig 1). In conjunction with our previous study [17], the data suggests that CANs are putative nociceptive neurons.

### RTX treatment

**Figure 2:**
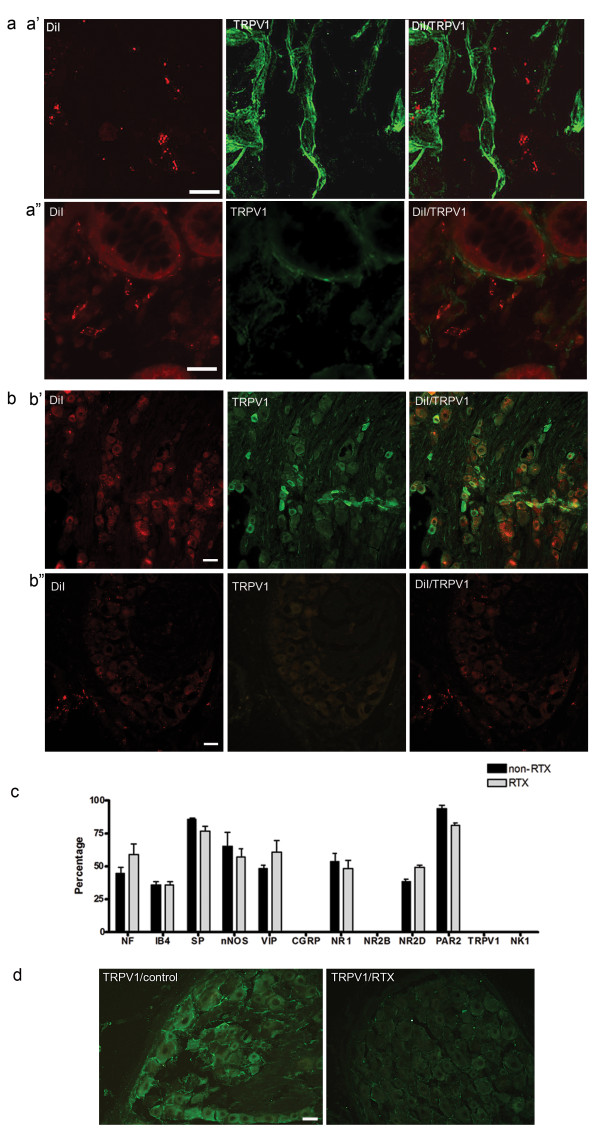
**Expression of the TRPV1 receptor is eliminated following treatment with RTX**. Photomicrographs of DiI and TRPV1 expression in control(a') and RTX(a") lesioned rats in submucosal plexus of the colon. RTX results in a decrease in the expression of TRPV1 labeling in the colon. Photomicrographs that show a decrease in both DiI and TRPV1 expression in lumbosacral DRG neurons(b") as compared to controls(b'). (c) Immunohistochemical characterization of CANs following RTX lesioning showed no change in peptide and receptor expression as compared to controls. RTX results in a decrease in the expression of TRPV1 labeling in thoracolumbar DRG neurons (e). Scale bars = 20 μm(a) and 50 μm(b, e).

Our data show that CANs are sensory neurons that may be involved in nociception. Therefore, we wanted to determine if CANs contribute to visceral hypersensitivity. As previously shown [17], CANs do not express TRPV1; therefore, we used an intrathecal and colonic injection of resiniferatoxin (RTX) to lesion TRPV1 expressing spinal primary afferent innervation to the colon. Immunohistochemical analysis revealed that all animals that received RTX (n = 50) had minimal to no TRPV1 receptors present in the colon (Fig 2a) or in DRG (L6-S1) neurons (Fig 2b). Immunohistochemistry analysis confirmed that RTX (n = 5) did not disrupt the peptide and receptor profile of CANs as compared to controls (n = 5) [17] (Fig 2c). Furthermore, the thoracolumbar (T13-L1) region of spinal cord has been implicated in visceral hypersensitivity [20-23]. Immunohistochemical analysis revealed that all animals that received RTX had minimal to no TRPV1 receptors present in the thoracic DRGs (T11-13) as compared to controls (Fig 2d).

### RTX decreases DRG afferent innervation of the colon

**Figure 3:**
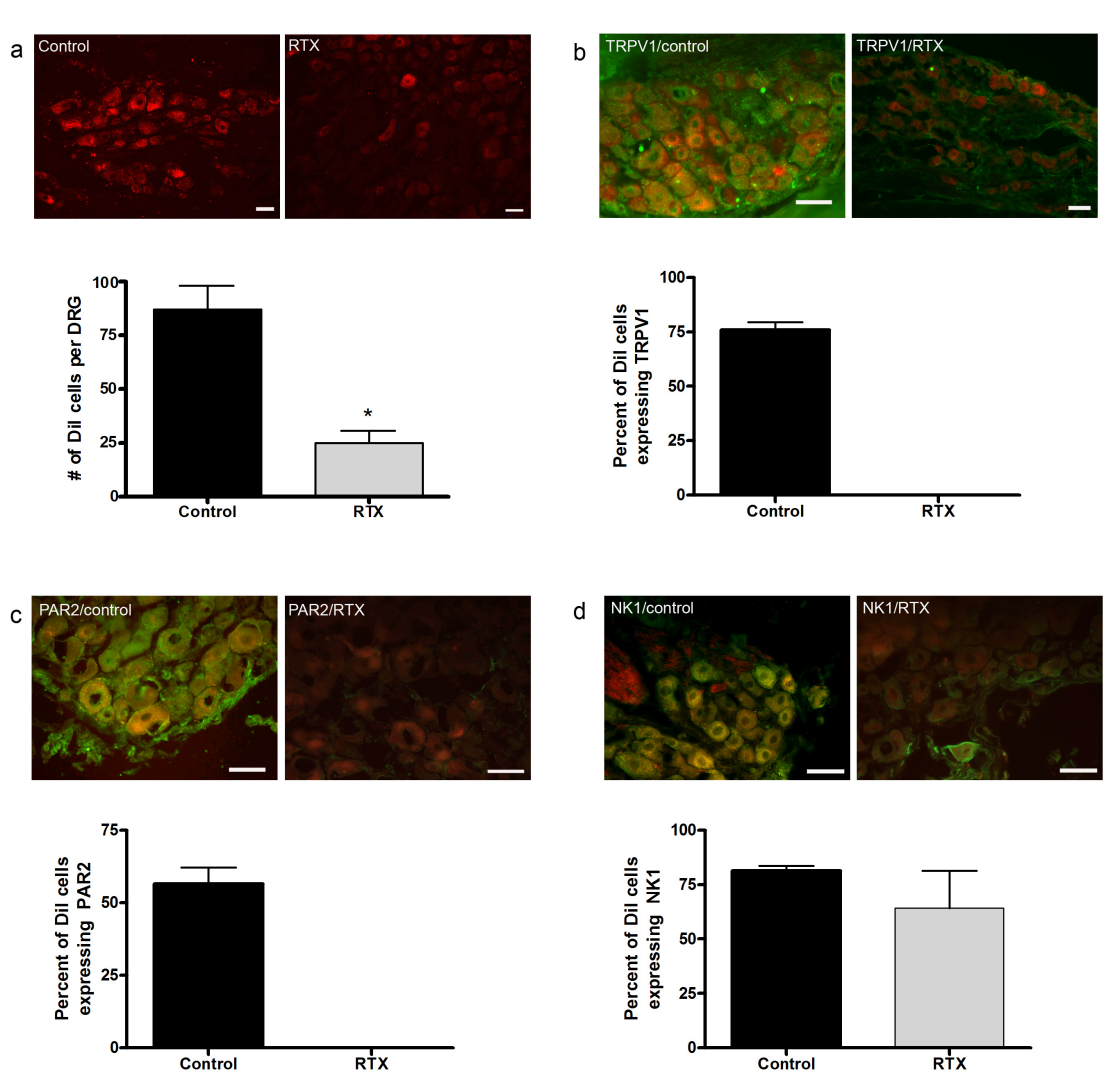
**Colonic innervation from lumbosacral DRG neurons is reduced following RTX treatment**. (a)Application of the retrograde tracer DiI into the colon wall revealed a 70% decrease in the number of DRG neurons that innervate colon as compared to controls (control 87 ± 12;RTX 26 ± 6;n = 5, p < 0.001). DiI neurons no longer co-localized with the TRPV1 receptor(b) (p < 0.0001) or the PAR2 receptor(c) (p < 0.0001) following RTX lesioning. However, there was no change in the expression of the NK1 receptor (d). Scale bars = 50 μm.

To verify the amount of remaining colonic innervation from DRG neurons following RTX, we injected DiI into the distal colon of both control (n = 5) and RTX animals (n = 5) (Fig 3a). Cell counts determined that 87 ± 12 total DRG neurons per lumbosacral DRG innervate the colon using this procedure. Following RTX treatment, we found 26 ± 6 DRG neurons per lumbosacral DRG innervate the colon. Thus, RTX treatment eliminates approximately two-thirds of visceral afferent innervation to the colon (p < 0.001) (Fig 3a). Since RTX treatment does not eliminate all DRG innervation to the colon, we performed immunohistochemistry to verify RTX did lesion all TRPV1 expressing neurons. Cell counts verified that the remaining DiI labelled DRG cells did not express TRPV1 (p < 0.0001) (fig 3b). We also determined that the remaining DiI labeled DRG neurons did not express the PAR2 receptor (p < 0.0001) (fig 3c). This finding is in agreement with data from Amadesi et al. [24] which demonstrated that the PAR2 receptor co-localized with TRPV1 receptors in DRG neurons. Further analysis revealed that the neurokinin-1 (NK1) receptor was still present on the remaining DiI neurons (fig 3d).

### Histopathology

**Figure 4:**
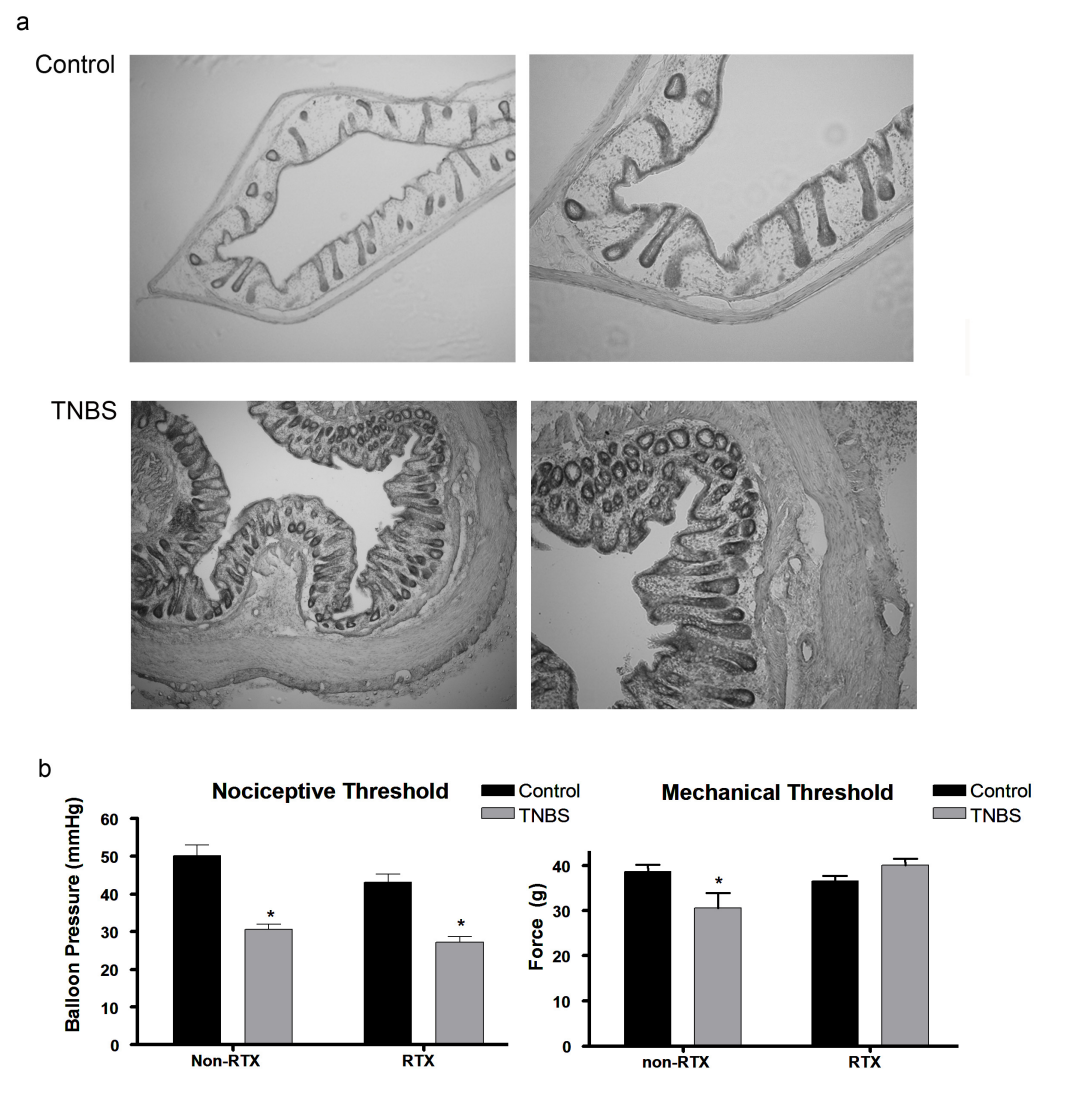
**TNBS induced visceral but not somatic hypersensitivity is still present following RTX treatment**. The presence of inflammation was seen in animals 14 days post-TNBS inflammation as shown by infiltration of neutrophils in the lamina propria, an increase in mast cells and mucosal ulceration (a). Fourteen days post-inflammation resulted in a decrease in balloon threshold for both Non-RTX and RTX animals. However, a decrease in peripheral mechanical threshold was seen in non-RTX animals but not RTX animals (b).

All rats that received TNBS enemas (n = 30) showed characteristics of inflammation: infiltration of neutrophils, an increase in the number of mast cells and ulceration of the mucosa 14 days post TNBS inflammation. Control animals showed a limited amount of neutrophil infiltration and no ulcerations of the mucosa. (Fig 4a).

### Behavior testing

We measured both visceral and peripheral mechanical hypersensitivity at 14 days post-TNBS inflammation. We observed a decrease in balloon pressure threshold for both non-RTX (n = 10; p < 0.001) and RTX TNBS animals (n = 10; p < 0.001) (Fig 4b). However, for peripheral mechanical threshold of the hindpaw we observed a decrease in threshold for non-RTX TNBS animals only (n = 10; p < 0.05), as compared to controls (n = 10; p > 0.05) (Fig 4b).

### TNBS-induced inflammation

**Figure 5:**
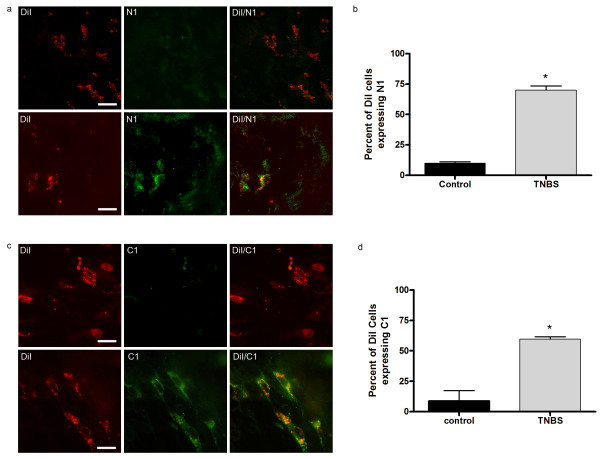
**NMDA receptor expression on CANs increases following TNBS-induced inflammation**. The NMDA receptor NR1 cassettes N1 (a) and C1(c) are up-regulated in DiI labelled neurons 14 days post-inflammation. N1 co-localization increased 50% (70% ± 3.3) as compared to controls (10% ± 1.145;p < 0.0001) (b). Where as C1 co-localization increased 51% (59.8% ± 1.7) as compared to controls (8% ± 8.6;p < 0.001) (d). Scale bars = 20 μm.

**Figure 6:**
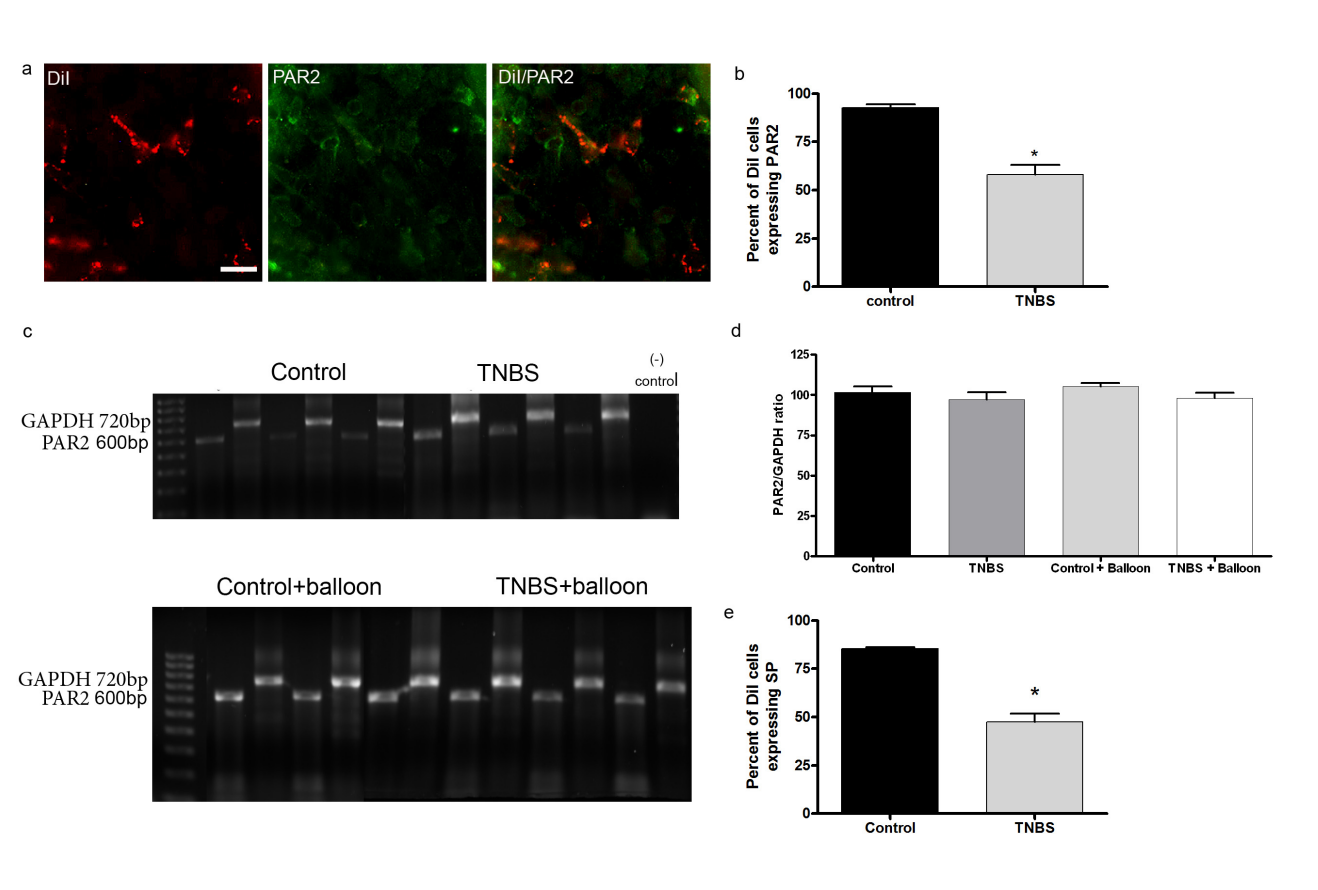
**Activation of the PAR2 receptor expressed on CANs following TNBS induced inflammation**. Approximately 40% of CANs expressing the PAR2 receptor are activated following inflammation as shown by an antibody that detects the N-terminal end of the PAR2 receptor (a, b) (p < 0.0003). Typical RT-PCR products for PAR2 (598 bp) and GAPDH (720 bp) (c). RT-PCR analysis revealed that mRNA expression of PAR2 does not change due to TNBS induced inflammation as compared to controls (c, d). A 60% decrease in the co-localization of SP expressed on CANs was seen following inflammation as compared to controls (p < 0.0008) (e). Scale bars = 20 μm.

As previously reported, CANs co-localize with the NR1 and NR2D subunits of the NMDA receptor as well as with the PAR2 receptor [17]. However, in control animals a minimal number of CANs co-localize with the N1 and C1 splice variants of the NR1 subunit of the NMDA receptor (Fig 5a, b). Fourteen days post-TNBS inflammation, both the N1 and C1 splice variants were up-regulated, a 50% (10% ± 1.145 control; 70% ± 3.3 TNBS) and 51% (8% ± 8.6 control; 59.8% ± 1.7 TNBS) increase in co-localization with CANs respectively (Fig 5). This is in agreement with another study that found an up-regulation of the NR1 splice variants in the colon following TNBS inflammation [12]. Furthermore, the co-localization of DiI with the PAR2 receptor appeared to decrease by approximately 50% following inflammation (P < 0.0003) (Fig 6a, b). The antibody raised against the PAR2 receptor recognizes the N-terminal end of the receptor or the un-activated form. A commercial antibody for the C-terminal end of the PAR2 receptor in rat is currently unavailable. We therefore used RT-PCR to detect PAR2 receptor mRNA, and found that the PAR2 receptor is not down-regulated following TNBS induced inflammation (Fig 6c, d). Therefore, the decrease in co-localization in CANs suggests the receptors were activated by proteases released from mast cells following inflammation of the colon. Immunohistochemical analysis determined, 14 days post-inflammation, co-localization of DiI labelled cells with SP decreased approximately 60% as compared to controls (P < 0.0008) (Fig 6e). The data suggests that following inflammation the PAR2 receptor expressed on CANs is activated thus causing the release of SP.

## Discussion

### CANs are putative nociceptors

The present results suggest that CANs are putative nociceptive neurons that may contribute to visceral hypersensitivity. We demonstrated that CANs co-localize with Na_v_1.9, a sodium channel that is present on nociceptive DRG neurons as well as enteric neurons [25,26]. Although the presence of Na_v_1.9 expressed on neurons does not determine if the neuron is a nociceptor, the presence of Na_v_1.9 is highly correlated with neurons that are found to be nociceptive [27]. Treatment with RTX resulted in the lesioning of TRPV1-expressing DRG neurons of the lumbosacral and thoracolumbar regions of the spinal cord. RTX also eliminated 70% of the lumbosacral DRG innervation to the colon. However, RTX treatment did not eliminate the visceral hypersensitivity response in TNBS treated animals. Although not definitive, the data suggests that CANs may contribute to visceral hypersensitivity. The visceral hypersensitivity observed seems to be associated with changes in both the NMDA and PAR2 receptors. We determined that the N1 and C1 cassettes of the NR1 subunit of the NMDA receptor are up-regulated in CANs 14 days following TNBS-induced inflammation. Also, TNBS inflammation causes the activation of the PAR2 receptors and this activation may cause the release of SP. Both these receptors have been implicated in mediating the visceral hypersensitivity response [27,28]. The present study demonstrates that CANs are putative nociceptive neurons that are involved in the plasticity of the colon following inflammation. This and our previous study [17] further suggest that sensory information can be transmitted from the gut to the spinal cord by CANs.

### Visceral hypersensitivity present following RTX treatment

Recently studies have suggested that the TRPV1 receptor is involved in the visceral hypersensitivity response [18,19]. However, our current study demonstrates that elimination of the TRPV1 receptor expressing neurons does not alter the visceral hypersensitivity response to colorectal distension (CRD). It is important to note that our study looks at visceral hypersensitivity 14 days following inflammation. Some studies looking at visceral hypersensitivity have only studied this effect using acute inflammatory agents that do not last longer than 8 days [29-31]. Conversely, Miranda et al. [32] demonstrated that application of a TRPV1 antagonist (JYL1421) 14 days following TNBS inflammation resulted in an attenuation of the visceral motor response (VMR) as compared to the TNBS-only group. However, the response was still present as compared to control responses. Thus, the data suggest that blocking the TRPV1 receptor does result in a decrease in visceral hypersensitivity but does not eliminate the response entirely. The authors hypothesized that TRPV1 may be involved in the initial inflammation response and hypersensitivity but not the maintenance of the response. Another study has implicated the TRPV1 receptor in motility. De Schepper et al. [33] used *In-Vivo *techniques in conjunction with capsazepine, a TRPV1 antagonist, to demonstrate that the sensitization of the TRPV1 receptor inhibits colitis-induced abnormal motility patterns. Therefore, it is plausible that the TRPV1 receptor is involved in an extrinsic reflex pathway modulating motility patterns during a state of inflammation. It has also been shown that this abnormal motility reflex was eliminated following ligation of the pelvic nerve [34]. In addition, Jones, III et al. [19] demonstrated that the TRPV1 receptor may be involved in non-inflammatory visceral hypersensitivity using a TRPV1 knock-out model. The authors demonstrated that TRPV1 -/- mice receiving zymosan and CRD did not show an increase in the VMR response; however, matched controls did have an increase in the VMR response. In this model zymosan did not induce inflammation, as shown by an MPO assay, which is in contrast with studies done in rats showing that zymosan causes inflammation [29]. Thus, our data in conjunction with the studies mentioned above suggest that the TRPV1 receptor may be involved in the mechanical component of the colonic inflammatory response but may not be the major component to maintaining visceral hypersensitivity.

Furthermore, it is important to note that the present study only looks at the involvement of the lumbosacral region of the spinal cord and its role in visceral hypersensitivity. Other studies have implicated the thoracolumbar region of the spinal cord in the mediation of the visceral hypersensitivity response [20-23,23]. Traub et al. [20] demonstrated that both lumbosacral and thoracolumbar regions of the spinal cord were activated during CRD as shown by cFos and cJun labeling. However, the sacral region of the spinal cord had a higher cFos and cJun response to CRD (80 mmHg) compared to the thoracic region. A later study indicated that there is a differential involvement of the two regions of the spinal cord following CRD [23]. To date, studies have not shown what receptors are involved in thoracolumbar mediation of visceral hypersensitivity and the exact role of this region in visceral hypersensitivity has yet to be determined. In the current study, we have shown that RTX lesions TRPV1 expressing DRG neurons in the thoracolumbar region in addition to the lumbosacral region following an intrathecal injection at the L6/S1 region of spinal cord and an intracolonic injection. Therefore, if the thoracolumbar DRG neurons are involved in visceral hypersensitivity, the present study demonstrates that the TRPV1 expressing neurons are not necessary to mediate visceral hypersensitivity.

### Up regulation of NMDA receptors

The NMDA receptor expressed on DRG neurons has commonly been thought to regulate visceral hypersensitivity. In addition, the NMDA receptor is expressed on enteric neurons of the colon in both rats and humans [35,36]. It was found that the NR2 subunits of the NMDA receptor, NR2B and NR2D, are present on colonic neurons [37]. Furthermore, Zhou et al. [12] showed the NR1 cassettes, N1 and C1, were up-regulated in a sub-set of myenteric neurons in the colon following TNBS-induced inflammation. In the present study we found similar results; however, we found that both the N1 and C1 cassettes were up-regulated in submucosal CANs. Studies have shown that when the N1 insert is present in NR1 the current amplitude is increased. This effect was shown to be potentiated by Zn^2+ ^and spermine [38,39]. Furthermore, Zheng et al [39] hypothesized that the increase in agonist-evoked currents may be a result of a conformational change induced by the expression of N1. This data suggests that an increase in NMDA receptor activity due to the increased expression of the N1 cassette on CANs may be contributing to the visceral hypersensitivity response observed following RTX and TNBS treatment. We also observed the presence of the C1 cassette on CANs following inflammation. The C1 insert is known to have an endoplasmic reticulum (ER) retention signal as well as four serines for phosphorlation. It is thought C1 is involved in the trafficking of newly formed NMDA receptors [40]. Therefore, the presence of both the N1 and C1 cassettes following inflammation could increase the activity of NMDA receptors on CANs causing neuroplasticity in the colon.

### Activation of PAR2

Recently it was suggested that activation of the PAR2 receptor is involved in inflammation-induced visceral hypersensitivity [16,41-43]. In the current study we demonstrated that the N-terminal end of PAR2 receptors on CANs was no longer present following TNBS-induced inflammation. It is known that proteases released in the colon during states of inflammation cleave the N-terminal end of the PAR2 receptor [41,44]. Our antibody for PAR2 was raised against the amino acid sequence on the N-terminal end of the PAR2 receptor just prior to the cleavage site; thus, the decrease in co-localization translates to an activation of the receptor. Previous studies looking at the role of proteases in IBS patients demonstrated that the activation of the PAR2 receptor can generate hypersensitivity symptoms in IBS patients [16]. In this study, colonic biopsies from IBS patients were found to have elevated levels of the proteases trypsin and tryptase as compared to non-IBS patients. A study using rats concluded that by administering the PAR2 specific agonist trypsin, hypersensitivity occurred in response to colorectal distention [15]. Hyun et al. [43] demonstrated that PAR2 -/- mice showed a decrease in signs of inflammation following TNBS-induced inflammation as shown by H&E staining, myeloperoxidase (MPO) activity, macroscopic damage score, and histological analysis. Furthermore, inflammatory mediators such as intracellular adhesion molecule-1 (ICAM-1) and vascular cell adhesion molecule (VCAM-1) were decreased and cyclooxygenase-1 (COX-1) was increased in PAR2-/- mice, suggesting that PAR2 receptors play a pro-inflammatory role. Previous studies suggest that activation of the PAR2 receptor causes the release of SP [41,42] SP is known to bind NK1 receptors in the dorsal horn of the spinal cord suggesting this interaction aids in mediating hypersensitivity[6]. Amadesi et al. [24] demonstrated that DRG neurons expressing the PAR2 receptor also express the TRPV1 receptor. The authors determined that activation of the PAR2 receptor caused release of SP following capsaicin injections. In the current study we demonstrated that lesioning of DRG neurons expressing TRPV1 receptors also eliminated expression of the PAR2 receptor in DRG neurons that innervate the colon. However, following inflammation, the remaining PAR2 receptors in the colon were activated and the visceral hypersensitivity response was present. Since we know that activation of the PAR2 receptor causes the release of SP, the data suggests that activation of PAR2 receptors on CANs may mediate the visceral hypersensitivity response via the release of SP acting on the remaining NK1 receptors expressed on DRG neurons. Further studies are needed to delineate the exact mechanism of this hypersensitivity response and whether SP is involved.

## Conclusion

In conclusion, the present study found that CANs are putative nociceptors as determined by the expression of Na_v_1.9 as well as other peptides and receptors commonly found on nociceptive neurons as shown previously [17]. We demonstrated that visceral hypersensitivity is still present in inflamed animals when TRPV1 expressing DRG neurons are eliminated. Following inflammation, CANs express the activated form of both NMDA and PAR2 receptors. The activation of these receptors may induce neuroplasticity in the colon which, in turn, may contribute to the visceral hypersensitivity response.

## Methods

### Animals

Experiments were performed on male Sprague-Dawley rats (n = 105) weighing 200-250 g. They were housed in pairs with free access to food and water in the University of Florida's animal care facility with a 12-h light/dark cycle. These facilities are AAALAC accredited. All experiments conformed to guidelines on the ethical use of animals as published by the International Association for the Study of Pain. All procedures were reviewed and approved by the University of Florida Institutional Animal Care and Use Committee.

### Resiniferatoxin (RTX) injections

Prior to injection, the animals were anesthetized with isoflurane (1-3% in O_2_). For intrathecal injections, an 18-gauge needle was used to make a lumbar puncture between vertebrae L1/L2. A small plastic tube (PE-10; Becton Dickinson, Sparks, MD) was inserted through the needle and the RTX solution (200 ng/25 μl in PBS) was injected slowly over 2 minutes. Following intrathecal injection, animals received an enema of RTX. A small plastic tube (PE-160; Becton Dickinson, Sparks, MD) was inserted 7 cm through the anus and the RTX solution (200 ng/500 μl) was infused slowly over 5 minutes. Animals were allowed to recover for two weeks prior to further procedures.

### 2,4,6-trinitrobenzene sulfonic acid (TNBS) enema

Prior to the enema, the animals were anesthetized with isoflurane (1-3% in O_2_). Animals received enemas of either TNBS (20 mg) in 50% ethanol and saline (1 ml) or saline alone (1 ml). A small plastic tube (PE-160; Becton Dickinson, Sparks, MD) was inserted through the anus 7 cm and the solution was infused slowly over 5 minutes.

### Laminectomy and DiI labeling

Prior to laminectomy, the animals were anesthetized with isoflurane (1-3% in O_2_). The dorsal portion of the L1 and/or L2 vertebrae was removed to expose the L6 and S1 segments of spinal cord. The dura covering the spinal cord was removed and a 5 × 2 mm piece of gel foam (Henry Schien, Melville, NY, USA) was placed onto the dorsal surface of the spinal cord. The gel foam was soaked in 40 μl of the retrograde tracer 1,1'-dioctadecyl-3,3,3',3'-tetramethyl-indo-carbocyanine perchlorate (DiI) (Molecular Probes, Eugene, OR, USA; 2.5 mg/ml in DMSO). The wound was closed by suturing in layers. A triple antibiotic was applied to the wound site and buprenorphine (0.3 mg/kg) was given i.m. twice a day for approximately 3 days after surgery.

### Laparotomy and DiI labeling of colonic DRG neurons

Prior to laparotomy, the animals were anesthetized with isoflurane (1-3% in O_2_). A small incision was made in the abdomen to expose the colon. Ten DiI injections (2 μl per injection; total 20 μl) were made in the wall of the colon using a Hamilton syringe, approximately 2 mm proximal to the bladder moving orally. The wound was closed by suturing in layers. A triple antibiotic was applied to the wound site and buprenorphine (0.3 mg/kg) was given i.m. twice a day for approximately 3 days after surgery.

### Behavior testing

For all behavioral testing, the researcher was blinded to all treatment groups.

#### Peripheral mechanical stimulation

Mechanical hypersensitivity was measured using an automatic Von Frey device (dynamic plantar aesthesiometer (Ugo Basile Biological Research Apparatus, Italy). Animals were placed within a plastic enclosure on a wire mesh floor. A computer-driven filament was extended up through the mesh floor and exerted an increasing amount of pressure (0-50 g) onto the hind paw. Both hind paws were tested. Mechanical pain threshold was determined by the amount of force required for the animal to withdraw its hind paw. The stimulus was repeated four times with 5 minute intervals and the mean pressures at the mechanical threshold were recorded for each rat.

#### Colonic distension

While anesthetized with isoflurane (1-3% O_2_) a balloon (7 cm in length) attached to small plastic tubing (PE-160; Becton Dickinson, Sparks, MD) was inserted through the anus and secured in place by taping the tubing to the base of the tail. Rats were placed in a plastic restraining device in order to prevent the animals from removing the balloon. The tubing was connected to a pressure transducer (Harvard Apparatus, Holliston, Massachusetts) to perform colorectal distension (CRD). Following recovery from anesthesia, the animals were allowed 10 minutes to acclimatize before behavioral testing began. The rats then received distension of the colon in 10 mmHg intervals (0-80 mmHg) until the first contraction of the testicles, tail, or abdominal musculature occurred. This is described as the visceral pain threshold indicative of the first nociceptive response. CRD was repeated four times with 5 minute intervals and the mean pressures at the nociceptive threshold were recorded for each rat.

### Perfusion fixation

After a survival time of 10 days (for surgery animals) or the end point of the experiment, animals were given a lethal dose of pentobarbital i.p. and perfused through the heart with cold 0.9% saline followed immediately with cold 4% paraformaldehyde in phosphate buffered saline (PBS). After fixation, the spinal cord, dorsal root ganglia (DRG) (L6-S1 and T11-L1) and the colon of the animal were removed. Tissue was then post-fixed in 4% paraformaldehyde in PBS for 24 hours at 4°C and then stored in 30% sucrose at 4°C for at least 24 hours.

### Hematoxylin and eosin staining

Colonic tissue from all behavioral animals was processed for histopathological evaluation. The tissue was processed using standard techniques for hematoxylin and eosin staining. The tissue was then evaluated for signs of colitis: infiltration of neutrophils in the lamina propria, an increase in mast cells and mucosal ulceration.

### Immunohistochemistry

Colonic tissue was sectioned at 20 μm and DRG tissue was sectioned at 10 μm on a cryostat, cut in 1:5 series sections, and air-dried for 1 hour. All preparations were washed 3 times (10 minutes each) in PBS, placed in blocking buffer containing 3% Normal Goat Serum (NGS) with PBS for 1 hour, and incubated in primary antibody in 3% NGS/0.3% tween-20/PBS (table 1) for 24 hours at 4°C. The sections were then washed 3 times in PBS (10 minutes each) followed by a 1 hour incubation in secondary antibody Alexa Fluro 488 (1:1000; Molecular Probes, Boston, MA) in 3%NGS/0.3% tween-20/PBS (table 2). Tissue was then washed 3 times (10 minutes each) and coversliped with ProLong^® ^Antifade Kit mounting media (Molecular Probes, Boston, MA) or Vectashield mounting media (Vector Laboratories, Burlingame, CA). The sections were visualized with filters for red and green excitation. Images were photographed on a Leica DM LB2 Fluorescence microscope (Leica, Wetzlar, Germany). Negative controls, where the secondary was applied to the sections in the absence of primary antibodies, were used to verify that non-specific binding of secondary antibodies to tissue did not occur. All images were processed using the Adobe Photoshop program. For colonic cell counts, at least a total of 100 cells per immunolabel were counted for analysis. For DRG cell counts, all DiI labelled cells were counted per DRG. Cell counts were done using the ImageJ program (NIH, Bethesda, MD) where DiI positive cells (in red) were counted and marked; double labelled cells were determined by superimposing the marked image with the label being investigated (in green).

**Table 1:** Primary antibodies used in immunohistochemistry of CANs.

**Label**	**Host**	**Dilution**	**Company**
NR1	Mouse	1:500	BD Biosciences

NR2B	Mouse	1:500	BD Biosciences

NR2D	Rabbit	1:100	Santa Cruz

NK1r	Mouse	1:500	Zymed

SP	Rabbit	1:250	Chemicon

PAR-2	Chicken	1:500	Aves Lab, Inc.

TRPV1	Rabbit	1:500	Affinity Bioreagents

CGRP	Rabbit	1:250	Courtesy of Dr. Michael Iadarola

nNOS	Rabbit	1:250	Chemicon

VIP	Rabbit	1:500	Chemicon

NF 200	Mouse	1:250	Sigma

IB4	Goat conjugated to IB4	1:1000	Invitrogen

N1	Rabbit	1:500	Courtesy of Dr. Michael Iadarola

C1	Rabbit	1:250	Courtesy of Dr. Michael Iadarola

Alexa Fluor 488	Goat anti-Rabbit	1:1000	Invitrogen

Alexa Fluor 488	Donkey anti-Mouse	1:1000	Invitrogen

Alexa Fluor 488	Goat anti-chicken	1:1000	Invitrogen

**Table 2:** RT-PCR primer sequences

**Primer name**	**Forward Sequence**	**Forward Temperature (°C)**	**Reverse Sequence**	**Reverse Temperature (°C)**	**Product Length (bp)**
PAR2	CACCAGTAAAGGGAGAAGTCT	59	GGGCAGCACGTCGTGACAGGT	59	598
GAPDH	CCTTCATTGACCTCAACTACATGGTCTA	63	TAGCCCAGGATGCCCTTT	55	720

### RT-PCR

Animals were euthanized with CO_2 _and the colon was quickly removed. Total RNA was isolated from colonic tissues using RNeasy Mini Kit from Qiagen (Valencia, CA, USA). Target transcripts were amplified with PCR primers from GenoMechanix (Gainesville, FL, USA) listed in Table 2. RT-PCR reactions were carried out using Access RT-PCR System from Promega (Madison, WI, USA) with the following cycle conditions: Initial denaturation at 95°C for 2 minutes; denaturing at 95°C for 30 seconds; annealing at 55°C for 1 minute and extension at 72°C for 2 minutes. A total of 32 cycles was used, followed by a final extension step of 72°C for 5 minutes. PCR products were separated on 1.2% agarose gel with 1× TBE buffer, viewed with ethidium bromide and analyzed with Bio-Rad Gel Doc EQ Gel Documentation System, Bio-Rad Laboratories (Hercules, CA, USA).

### Statistical analysis

All statistics were run using Prism GraphPad version 6. For analysis of co-localization an unpaired t-test was used. For analysis of behavioral testing a two-way analysis of variance (ANOVA) followed by Bonferroni posttest was used for each time point. A one-way ANOVA was used to analyze RT-PCR data. All values are expressed as means ± SEM. Data sets were considered significant for P < 0.05.

## Competing interests

The authors declare that they have no competing interests.

## Authors' contributions

SKS participated in the design of the study, carried out all of the experiments and analyses and helped write the manuscript. RMC guided the design of the study and helped with writing the manuscript. All authors read and approved the final manuscript.
